# Improving phytosterol biotransformation at low nitrogen levels by enhancing the methylcitrate cycle with transcriptional regulators PrpR and GlnR of *Mycobacterium neoaurum*

**DOI:** 10.1186/s12934-020-1285-8

**Published:** 2020-01-28

**Authors:** Yang Zhang, Xiuling Zhou, Xuemei Wang, Lu Wang, Menglei Xia, Jianmei Luo, Yanbing Shen, Min Wang

**Affiliations:** 1 Key Laboratory of Industrial Fermentation Microbiology, Ministry of Education, Tianjin Key Laboratory of Industrial Microbiology, College of Biotechnology, Tianjin University of Science & Technology, Tianjin, 300457 China; 2 College of Life Science, Liaocheng University, Liaocheng, 252059 Shandong China

**Keywords:** *Mycobacterium neoaurum*, Androstenedione, Methylcitrate cycle, Transcriptional regulator, Nitrogen level

## Abstract

**Background:**

Androstenedione (AD) is an important steroid medicine intermediate that is obtained via the degradation of phytosterols by mycobacteria. The production process of AD is mainly the degradation of the phytosterol aliphatic side chain, which is accompanied by the production of propionyl CoA. Excessive accumulation of intracellular propionyl-CoA produces a toxic effect in mycobacteria, which restricts the improvement of production efficiency. The 2-methylcitrate cycle pathway (MCC) plays a significant role in the detoxification of propionyl-CoA in bacterial. The effect of the MCC on phytosterol biotransformation in mycobacteria has not been elucidated in detail. Meanwhile, reducing fermentation cost has always been an important issue to be solved in the optimizing of the bioprocess.

**Results:**

There is a complete MCC in *Mycobacterium neoaurum* (MNR), *prpC*, *prpD* and *prpB* in the *prp* operon encode methylcitrate synthase, methylcitrate dehydratase and methylisocitrate lyase involved in MCC, and PrpR is a specific transcriptional activator of *prp* operon. After the overexpression of *prpDCB* and *prpR* in MNR, the significantly improved transcription levels of *prpC*, *prpD* and *prpB* were observed. The highest conversion ratios of AD obtained by MNR-*prpDBC* and MNR-*prpR* increased from 72.3 ± 2.5% to 82.2 ± 2.2% and 90.6 ± 2.6%, respectively. Through enhanced the PrpR of MNR, the in intracellular propionyl-CoA levels decreased by 43 ± 3%, and the cell viability improved by 22 ± 1% compared to MNR at 96 h. The nitrogen transcription regulator GlnR repressed *prp* operon transcription in a nitrogen-limited medium. The *glnR* deletion enhanced the transcription level of *prpDBC* and the biotransformation ability of MNR. MNR-*prpR*/Δ*glnR* was constructed by the overexpression of *prpR* in the *glnR*-deleted strain showed adaptability to low nitrogen. The highest AD conversion ratio by MNR-*prpR*/Δ*glnR* was 92.8 ± 2.7% at low nitrogen level, which was 1.4 times higher than that of MNR.

**Conclusion:**

Improvement in phytosterol biotransformation after the enhancement of propionyl-CoA metabolism through the combined modifications of the *prp* operon and *glnR* of mycobacteria was investigated for the first time. The overexpress of *prpR* in MNR can increase the transcription of essential genes (*prpC, prpD and prpB*) of MCC, reduce the intracellular propionyl-CoA level and improve bacterial viability. The knockout of *glnR* can enhance the adaptability of MNR to the nitrogen source. In the MNR*ΔglnR* strain, overexpress of *prpR* can achieve efficient production of AD at low nitrogen levels, thus reducing the production cost. This strategy provides a reference for the economic and effective production of other valuable steroid metabolites from phytosterol in the pharmaceutical industry.

## Background

Steroid drugs are widely used in anti-inflammation, endocrine regulation, and fertility management [1]. The global market for steroidal drugs has exceeded $10 billion, and the industry prospects are extensive [2]. Microbial biotransformation using phytosterols as substrates has become an important way to produce steroid precursors, such as androstenedione (AD) and 9α-hydroxyandrostenedione (9α-OH-AD) [3]. Phytosterols mainly exist in plants and are abundant in crops, such as beans and cereals, which are starting materials for steroid drugs production through mycobacterium transformation because of their low cost and environment-friendly attributes [4]. The gene-editing of the sterol side chain degradation metabolic pathway has made the industrial production of the steroidal pharmaceutical precursor by phytosterol biotransformation possible [5–11], but on the premise of improving production efficiency, reducing production costs is still the continuous pursuit of industrial production. Recently, studies on toxic metabolites, such as reactive oxygen species (ROS) in the production process of strains, have provided new ideas for the study of phytosterol conversion [12, 13].

The production of AD and 9α-OH-AD from phytosterol bioprocessing mainly involves the degradation of aliphatic side chains through *β*-oxidation, and several propionyl-Coenzyme A (CoA) and acetyl-CoA are produced [14–16]. For example, when β-sitosterol is used as the substrate, the complete degradation of the side chain will produce three molecules of propionyl-CoA and one molecule of acetyl-CoA [17]. Propionyl-CoA plays an essential role in maintaining the balance of metabolic flow and energy supply during the metabolism of sterols and odd-chain fatty acids in mycobacteria [18]. Previous studies have shown that the excessive accumulation of intracellular propionyl-CoA in *Mycobacterium smegmatis* and *M. tuberculosis* produces a toxic effect, which seriously affects the growth of mycobacteria on propionate or mixtures of propionate and glucose [19, 20]. Three different detoxification mechanisms in mycobacteria have been proposed, namely, the methylmalonyl cycle pathway (MMC), the 2-methylcitrate cycle pathway (MCC), and the incorporation of propionyl moieties into cell envelope methyl-branched lipids [20]. Our recent research shows that enhanced MMC in *Mycobacterium neoaurum* (MNR) promotes the conversion of propionyl-CoA to nontoxic succinyl-CoA and improves the transformation of phytosterols [21]. Therefore, while metabolizing propionyl-CoA, MCC also has the dual effects of detoxification and energy supply.

Previous findings have shown that in the MCC of mycobacteria, propionyl-CoA is converted to pyruvate at a molar ratio of 1:1 [22, 23]. The methylcitrate synthase (MCS or PrpC; encoded *prpC*) catalyzes the condensation of propionyl-CoA with oxaloacetate to 2-methylcitrate, which is then dehydrated to produce methylaconitate. This step is accomplished by either methylcitrate dehydratase (MCD or PrpD; encoded *prpD*) or by the combined activity of methylcitrate dehydratase (AcnD) and methylaconitate cis–trans isomerase (PrpF). Following dehydration, methylaconitate is rehydrated by aconitase (AcnB) to yield methylisocitrate and cleaved by methylisocitrate lyase (MCL or PrpB; encoded *prpB*) or by isocitrate lyase to produce pyruvate and succinate [24]. The *prp* operon plays a key role in the assimilation of propionyl-CoA in *Mycobacterium tuberculosis* [25]*,* and *Mycobacterium smegmatis* [19] to obtain carbon and energy and prevents the accumulation of toxic metabolites. Masiewicz et al. [26] identified the novel transcription factor (PrpR; encoded *prpR*) that regulates the MCC by induces self-transcription and activating the *prp* operon in *M. tuberculosis*. PrpR is essential for the utilization of odd-chain-length fatty acids, and the *prpR* knockout strain exhibited an inhibited growth on propionate as a sole carbon source. Therefore, speculation is that the effector of PrpR may be propionyl-CoA, which is an intermediate metabolite of both odd-chain-length fatty acids and propionate.

Current research on MCC in mycobacteria is focused on *Mycobacterium tuberculosis* and *Mycobacterium smegmatis*, which are mainly related to the function of MCC at low cholesterol concentrations and other substrates. The effect of MCC on the transformation ability of steroid precursor production strains at a high concentration of phytosterol has not been reported, which is worthy of further study.

Nitrogen is an essential element for the microbial synthesis of proteins, nucleic acids, and substances required for growth. The supply of nitrogen sources strongly influences the growth and metabolism of heterotrophic microorganisms and autotrophic algae, which is very important for the efficient generation of target products during fermentation [27–30]. The nitrogen sources that can be absorbed and utilized by microorganisms in the fermentation industry are usually expensive yeast extract and peptone [31]. Therefore, finding an inexpensive alternative nitrogen source is the primary method to reduce the cost of the medium [32]. We have tried to use the hydrolysate of mycobacterial cells instead of yeast extract to reduce the cost of nitrogen source [21], but the relatively complex process limits its large-scale application in industrial production. Compared with the use of cheap nitrogen sources, improving the adaptability of strains to poor nitrogen sources is more direct and effective in reducing the cost of nitrogen sources.

Considerable research has shown that the utilization of nitrogen sources by microorganisms is controlled by regulatory proteins such as GlnR [33]. Studies on mycobacteria have shown that GlnR can regulate nitrogen assimilation in response to its limited availability [34]. GlnR is a conserved OmpR-like transcription factor, which is one of the regulators of nitrogen metabolism in actinomycetes, can regulate more than 100 genes, and has a wide range of effects on the growth and adaptation environment [35]. In a recent study, Liu et al. [36] found that GlnR directly binds to the promoter region of *prpDBC* and inhibits its transcription in *M. smegmatis*. However, little is known about the effects of regulatory factors, PrpR and GlnR, on the transformation of phytosterols by mycobacteria. Improving the nitrogen source adaptation level of a strain by nitrogen regulation factors is significant, and to the best of our knowledge, this process has not been reported.

In the present work, we show for the first time that genetically manipulating transcription factors can improve the AD production in a *Mycobacterium*. The transcriptional level of *prpDBC* and the changes in cell growth and AD production after the overexpression of *prpR* in MNR were studied. The effects of *glnR* deletion on the transcription level of *prpDBC* and the AD molar conversion rates were investigated at different nitrogen source levels. To further improve the productivity of AD, a *prpR* overexpressing strain MNR-*prpR*/Δ*glnR* was constructed under *glnR* deletion, and the AD productivity of MNR-*prpR*/Δ*glnR* at different nitrogen source levels was evaluated. In summary, strategies for effectively enhancing AD production by using *prp* operon and *glnR* were first reported.

## Materials and methods

### Mutant strains construction

All modified strains and plasmids used in this work are listed in Table 1. *Escherichia coli* DH5α was used for plasmid replication. Genes overexpression and deletion methods have been reported in previous studies [21]. In MNR, the vector pMV261 having kanamycin resistance (Kan^R^) was used to overexpress the target gene. Genes of PrpR and PrpDBC were amplified from the genome of MNR. The *prpR* and *prpDBC* genes were recombined with the linearized pMV261 using the In-Fusion HD Cloning method to generate recombinant plasmids, which were named pMV261*-prpR* and pMV261*-prpDBC*, respectively. The recombinant plasmid introduced to MNR through electroporation to gain the recombinant strains (MNR-*prpR* and MNR-*prpDBC*). The empty plasmid control strain (MNR-pMV261) was constructed by the introduction of pMV261 into the MNR. The in-frame deletion mutants of *glnR* were built by referring to the methods of Yao et al. [22]. The 1131 bp upstream sequence and 1107 bp downstream sequence of *glnR* were obtained as a recombinant fragment by PCR. Two fragments were ligated into the plasmid p2NIL and then digested with *Pac*I and ligated with a selection marker cassette from pGoal19 to construct a homologous recombinant plasmid. The constructed plasmid was transferred into mycobacterial cells by electroporation, and the *glnR* knockout strain was screened according to the previously reported protocol [11]. Construction of recombinant strains MNR-*prpR*/Δ*glnR* and MNR-*prpDBC*/Δ*glnR* by electroporation of plasmids pMV261*-prpR* and pMV261*-prpDBC* into MNR*ΔglnR*.

**Table 1:** Strains, plasmids, and primers used in this study

Strains, plasmids, and primers	Significant properties	Source or purpose
Strains		
* E. coli* DH5α	General cloning host	Transgen Biotech
* Mycobacterium neoaurum* TCCC 11,978 (MNR)	Wild type	Tianjin University of Science and Technology Culture Collection Center (TCCC)
MNR-prpR	*prpR* overexpressed strain of MNR	This work
MNR-prpDBC	prpDBC overexpressed strain of MNR	This work
MNRΔglnR	Deletion of *glnR* in MNR	This work
MNRΔglnR::glnR	*glnR* complemented in MNRΔglnR	This work
MNR-prpR/ΔglnR	*prpR* over-expressed strain of MNRΔglnR	This work
MNR-prpDBC/ΔglnR	*prpDBC* over-expressed strain of MNRΔglnR	This work
MNR-pMV261	MNR carrying vector pMV261	This work
Plasmids		
pMV261	Shuttle vector of *mycobacterium* and *E. coli*, carrying the heat shock *hsp60* promoter, *Kan*^*R*^	Dr. W. R. Jacobs Jr
p2NIL	Plasmid for allelic exchange, non-replicative in *Mycobacterium* species, *Kan*^*R*^	Dr. T. Parish
pGOAL19	*lacZ*, *hyg* and *sacB* marker genes cassette-containing vector, *Hyg*^*R*^	Dr. T. Parish
pMV261*-prpR*	pMV261 carrying an extra *prpR* for overexpression, *Kan*^*R*^	This work
pMV261*-prpDBC*	pMV261 carrying an extra *prpDBC* for overexpression, *Kan*^*R*^	This work
pMV261*-glnR*	pMV261 carrying an extra *glnR* for overexpression, *Kan*^*R*^	This work
pKO-*glnR*	p2NILcarrying the homologous arms of *glnR* and the selection markers from pGOAL19	This work
Primers		
PCR for overexpression		
* prpR*-f	GGATCCAGCTGCAGAATTCGTGGCGAAGACATTCGCGGG	*prpR* amplification
* prpR*-r	CGCTAGTTAACTACGTCGACTCAGTTGGCGGGCGGGTAGG	*prpR* amplification
* prpDBC*-f	GGATCCAGCTGCAGAATTCGTGCGGATCATGCAGAAACA	*prpDBC* amplification
* prpDBC*-r	CGCTAGTTAACTACGTCGACTCACCGGCGACGTTCCAGCG	*prpDBC* amplification
PCR for deletion		
* glnR*-U-f	ATAAACTACCGCATTAAAGCTTTCTGAACCCGTCCAGGTCGA	*glnR* deletion
* glnR*-U-R	CTCGGGACCGACGATCGCGATATCGGCG	*glnR* deletion
* glnR*-D-f	ATCGTCGGTCCCGAGTACGAGGCGCTG	*glnR* deletion
* glnR*-D-R	TGACACTATAGAATACATAGGATCCGGTCCGCGGACGGAAAGTGA	*glnR* deletion
For qRT-PCR		
* 16 s*-f-RT/*16 s*-r-RT	GTAGGGTCCGAGCGTTGTC/GCGTCAGTTACTGCCCAGAG	Quantitative RT-PCR
* prpR*-f-RT/*prpR*-r-RT	CCTGGGAACCCCGAAGA/GCCCGACATAGGGAAACG	Quantitative RT-PCR
* prpD*-f-RT/*prpD*-r-RT	TGGTCAAGGGCATCTGTCTG/TGCCCGATGGCGTTGTA	Quantitative RT-PCR
* prpB*-f-RT/*prpB*-r-RT	GACCAGGTGAACCCCAAGC/GCGGGCACAGATCACAAAG	Quantitative RT-PCR
* prpC*-f-RT/*prpC*-r-RT	GCCCAATCTGGATTTCCC/AGCGTTTGAAGCGGTTTG	Quantitative RT-PCR
* glnR*-f-RT/*glnR*-r-RT	CCTCGGGGAGTTGGTCAT/ GTTGGGCCAGATACTTCAGC	Quantitative RT-PCR

### Medium and cultivation conditions

All reagents and substrates were prepared as previously described [13, 21]. *E. coli* DH5*α* was cultured at 37 °C in Luria–Bertani (LB) medium (10.0 g/L tryptone, 5.0 g/L yeast extract, and 10 g/L NaCl at pH 7.0), where appropriate, kanamycin was added to the medium with a final concentration of 50 μg/mL. Mycobacteria seeds were prepared according to the previous description [21]. Seeds were inoculated into a shake flask containing 50 mL of fermentation medium at 10% inoculation for phytosterol bioconversion and then cultured for 168 h at 30 °C and 200 rpm. The phytosterol biotransformation medium (pH 7.5) contained 10 g/L glucose, 2.0 g/L (NH_4_)_2_HPO_4_, 0.05 g/L ferric ammonium citrate, 0.25 g/L MgSO_4_, 25 mM hydroxypropyl-β-cyclodextrin, and 5 g/L phytosterol. The normal nitrogen source level was set to 1 (N^1^), and one-tenth (N^0.1^), one-half (N^0.5^), three-fifths (N^0.6^), seven-tenths (N^0.7^), four-fifths (N^0.8^), and nine-tenths (N^0.9^) of its weight were added to the fermentation medium to investigate the effects of different nitrogen source levels on the strain.

### Quantitative reverse transcription-PCR (qRT-PCR)

For qRT-PCR analysis, the cells were cultured for 48 h and collected by centrifugation at 8,000×*g* for 10 min at 4 °C. Isolation of RNA was carried out according to the method described by our previous description [13]. The qRT-PCR analysis was performed according to the previously described method [37]. The primers for qRT-PCR were listed in Table 1. The messenger RNA (mRNA) level of the 16S rRNA gene was used as the housekeeping gene (internal control) to normalize the sampling errors [38]. Relative gene expression levels were calculated by the comparative Ct method (2^−ΔΔCt^ method) [39].

### Determination of intracellular propionyl-CoA concentrations

During the fermentation of MNR, MNR-*prpR* and MNR-*prpDBC*, samples were taken at 24-hour intervals, and the samples were divided equally. One part was used to detect the dry cell weight (DCW) according to the previous method [40], and the other was used to analyze intracellular propionyl-CoA. Intracellular propionyl-CoA levels were detected using a modified method previously described by Xu et al. [41]. Cells cultures were harvested and quenched in liquid nitrogen and washed twice with precooled phosphate-buffered saline (PBS, pH 8.0), then added with lysis buffer (10% trichloroacetic acid and 2 mM dithiothreitol), and repeatedly frozen and thawed four times using liquid nitrogen ice water, then sonicated on ice for 5 min (work 5 s, stop 5 s) using an ultrasonic disintegrator (JY96-IIN; Ningbo Xinzhi Instruments Inc., Ningbo, China). The supernatant was collected by centrifugation at 14,000×*g* for 10 min at 4 °C and transferred to an equilibrated solid-phase extraction column (Sep-Pak, tC18; Waters, Milford, MA). After loading the sample, wash the extraction column with 0.1% trifluoroacetic acid (TFA). The extraction column was subsequently eluted with 40% acetonitrile containing 0.1% TFA. The collected eluent was freeze-drying performed in a freeze-dryer (Alpha 2–4 LD plus, Martin Christ, Osterode am Harz, Germany) and stored at − 80 °C until analysis.

For the analysis of propionyl-CoA, the prepared samples were assayed by high-performance liquid chromatography (HPLC) using Agilent 1260 (Agilent Technologies, Santa Clara, CA, U.S.A.) equipped with UV detection at 260 nm. HPLC analysis was realized on a reversed-phase C18 column (250 mm × 4.6 mm) at 25 °C, and the two mobile-phase solvents used were buffer A (acetonitrile) and buffer B (100 mM ammonium acetate, pH 5.8). The linear gradient elution was carried out at 0.8 mL/min, and elution condition was as follows: 2–12% A (0–5 min), 12–38% A (5–15 min), 38% A (15–17 min), 38–2% A (17–19 min), data collection was stopped at 30 min. The content of propionyl-CoA was calculated by the external standard method.

Propionyl-CoA (Sigma, USA) was used for the preparation of the standard curve. The standard stock solution of propionyl-CoA (100 μmol/L) was prepared and stored at − 80 °C. Propionyl-CoA standard working solutions were prepared by diluting the stock solutions to 0.01, 0.05, 0.1, 0.2, 0.5, 1, 2, 2.5, 5.0, and 10 μmol/L. The calibration curve was prepared using the standard working solutions of propionyl-CoA. For each sample, the propionyl-CoA concentration was calculated by interpolating the sample measurement in the standard curve. Finally, the intracellular propionyl-CoA concentrations were calculated according to the DCW value of each sample.

### Detection of bacterial viability and product analysis

The bacterial viability using the modified CCK-8 method was determined as described previously [13]. The fermentation broth adjusts the OD_600_ value to 1 using a Tris–HCl buffer (pH 7.2). Add 190 μL fermentation broth and 10 μL of WST-8 to the 96-well plate, detect absorbance at 450 nm after 1 h incubate at 30 °C. For product analysis, samples were taken every 24 h during the conversion of phytosterol, and the same volume of ethyl acetate was added to 1 mL fermentation broth for 30 min. After 12,000×*g* centrifugation for 10 min, 200 μL extract was dissolved in 80% methanol after vacuum drying and centrifuged at 12,000 ×* g* for 20 min for HPLC analysis. HPLC analysis was prepared according to the method described by the previous description [21].

## Results and discussion

### Sequence homology and function analysis of PrpR, PrpD, PrpB, PrpC and GlnR

**Fig. 1:**
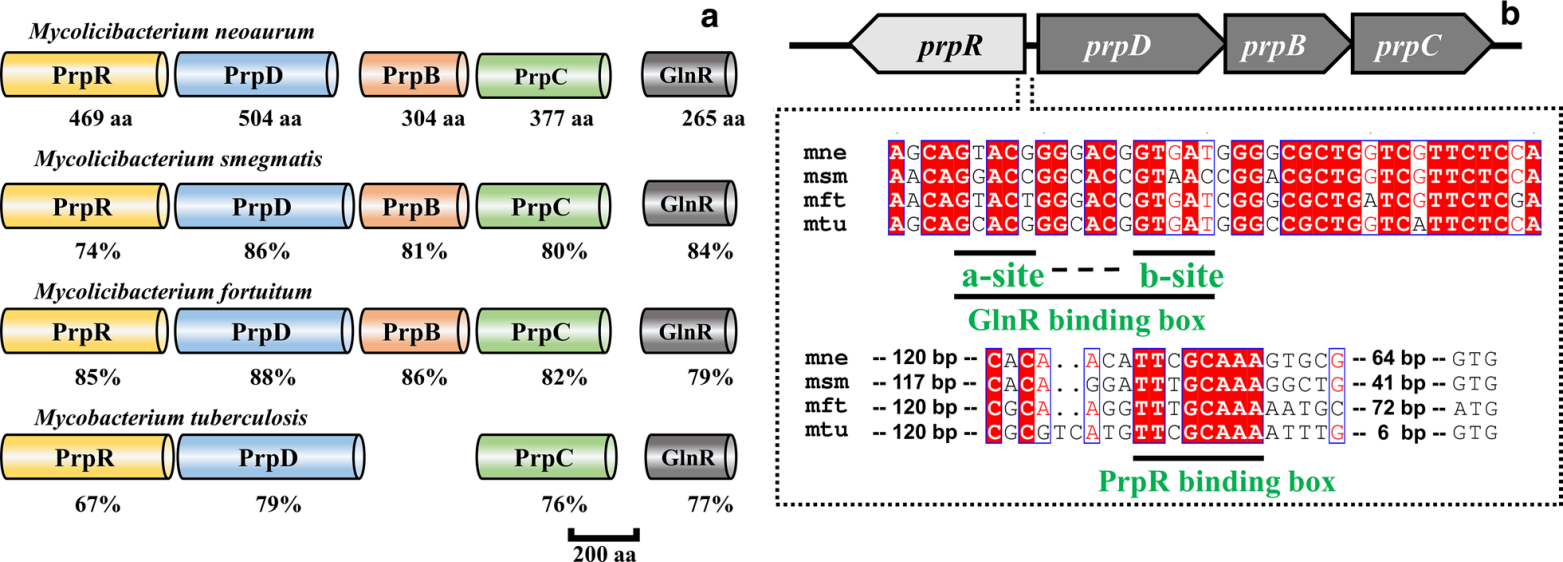
Sequence homology and function analysis of PrpR, PrpD, PrpB, PrpC and GlnR of four mycobacteria. **a** The structural variations of the PrpR, PrpD, PrpB, PrpC and GlnR. **b** GlnR and PrpR bind the promoter region of *prpDBC* in *M. neoaurum*

Previous studies confirm that *M. smegmatis* contains *prpD*, *prpD* and *prpC*, but *M. tuberculosis* contains only *prpD* and *prpC*. The activity of PrpB is provided by isocitrate lyases of *M. tuberculosis* (ICL1 and ICL2; encoded *icl1* and *icl2*), which are two isoforms involved in the glyoxylate pathway. The amino acid sequence analysis showed that the PrpD, PrpB and PrpC of MNR were similar to *M. smegmatis* and *M. tuberculosis* with sequence identity between 76 and 88% (Fig. 1a). The sequence concordance between the PrpR of MNR and the transcription factor PrpR of *M. smegmatis* was 74%. Sequence homology alignment results showed that MNR and 9a-OH-AD producing strain *M. fortuitum* (MFT) [13] had a complete MCC, and the similarity of each enzyme was higher than 82%. Therefore, MCC is widely present in mycobacteria with phytosterol transformation ability. The analysis of the *prpDBC* upstream region revealed a typical PrpR binding motif (TTCGCAAA), and GlnR binding motif (GGACCGGCACCGTAAC) were observed in the upstream region of *prpDBC* in MNR (Fig. 1b). According to Liu et al. [36], GlnR directly binds to the promoter region of *prpDBC* and inhibits its transcription in *M. smegmatis*. So the same mechanism also exists in MNR.

### The function of prp gene cluster in MNR

#### PrpR promotes the transcription of *prpDBC* in MNR

**Fig. 2:**
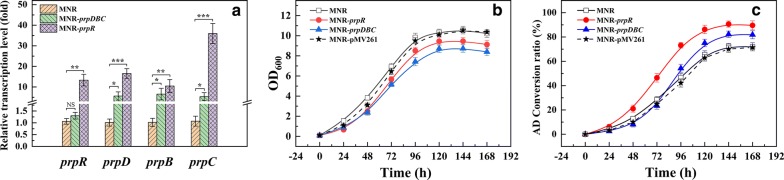
Recombinant strain qRT-PCR analysis, growth status, and AD conversion ratio of MNR, MNR-*prpR* and MNR-*prpDBC*. **a** qRT-PCR analysis of the expression variations of *prpR*, *prpD*, *prpB* and *prpC*. **b** Growth curves of MNR, MNR-*prpR*, and MNR-*prpDBC*. **c** Time courses of biotransformation rates of AD by MNR, MNR-*prpR*, and MNR-*prpDBC*. Error bars indicate the standard deviation from three independent experiments and the levels of statistical significance are indicated as follows: *p < 0.05; **p  < 0.01; ***p < 0.001; NS, indicates no significant difference (p > 0.05)

The *prpR* and *prpDBC* overexpression strains (MNR-*prpR* and MNR-*prpDBC*) were constructed for studying the effect of PrpR on *prpDBC* in MNR. The relative transcription levels of *prpD*, *prpB* and *prpC* in MNR, MNR-*prpR* and MNR-*prpDBC* were compared in the presence of phytosterols. As shown in Fig. 2a, the relative transcription level of *prpDBC* increased significantly in the *prpR*-overexpressed strain compared with that in the wild type strain. The *prpD*, *prpB* and *prpC* transcripts increased in MNR-*prpR* 16.5 ± 2.4-, 10.5 ± 3.1- and 36.0 ± 4.9-fold*,* respectively. The overexpression of *prpDBC* increased the transcription levels of *prpD*, *prpB* and *prpC* 5.6 ± 2.0-, 6.5 ± 2.8- and 5.3 ± 2.0-fold, respectively. Therefore, *prpR* is a positive transcription regulator of *prpDBC* in MNR. In addition, the relative transcription level of *prpD*, *prpB* and *prpC* in strain MNR-*prpR* was higher than that in MNR-*prpDBC*. The reason may be that the PrpR protein produced by overexpression of *prpR* in MNR-*prpR* can promote the transcription of *prpD*, *prpB* and *prpC* on the genome, and also promote the transcription of *prpR* gene, thus producing a dual promoting effect on *prpD*, *prpB* and *prpC* transcription. Therefore, as far as the relative transcription level of *prpD*, *prpB* and *prpC* is concerned, overexpression of *prpR* alone has a better effect.

#### Enhancement of AD productivity by overexpressing the *prp* operon

The effects of *prp* operon enhancement on cell growth and AD production were studied. It was found that the conversion rate of a single gene (*prpD*, *prpB* and *prpC*) overexpression was always lower than that of co-expression of three genes (data not shown). Therefore, the *prpR* and *prpDBC* overexpression strains (MNR-*prpR* and MNR-*prpDBC*) were selected for comparative study with the parental strain (MNR) and the control strain (MNR-pMV261). Although all strains have the same growth trend, the introduction of plasmids could reduce the biomass, which is more pronounced when overexpressing foreign genes. The biomass of the MNR-*prpR* and MNR-*prpDBC* were lower than the MNR and control strain, and MNR-*prpR* had the smallest biomass (Fig. 2b). As shown in Fig. 2c, the AD production capacity of recombinant strains was higher than that of MNR and MNR-pMV261. The highest AD molar conversion rates of MNR-*prpR* and MNR-*prpDBC* were 90.6 ± 2.6% and 82.2 ± 2.2%, respectively, which were higher than the parental strain (72.3 ± 2.5%) and control strain (70.1 ± 2.7%). The AD conversion rate of MNR-*prpDBC* was always lower than that of MNR and MNR-pMV261 before 48 h, and the conversion rate of MNR-pMV261 was consistently lower than that of MNR. The main reason for this phenomenon may be due to the increased metabolic load caused by the generation of antibiotic resistance and genes overexpression. The above results showed that the overexpression of *prp* operon enhanced the AD production capacity of MNR, and the effect of *prpR* overexpression was most evident.

#### Enhancement of the MCC can reduce the accumulation of propionyl-CoA in cells and increase the viability of bacteria

**Fig. 3:**
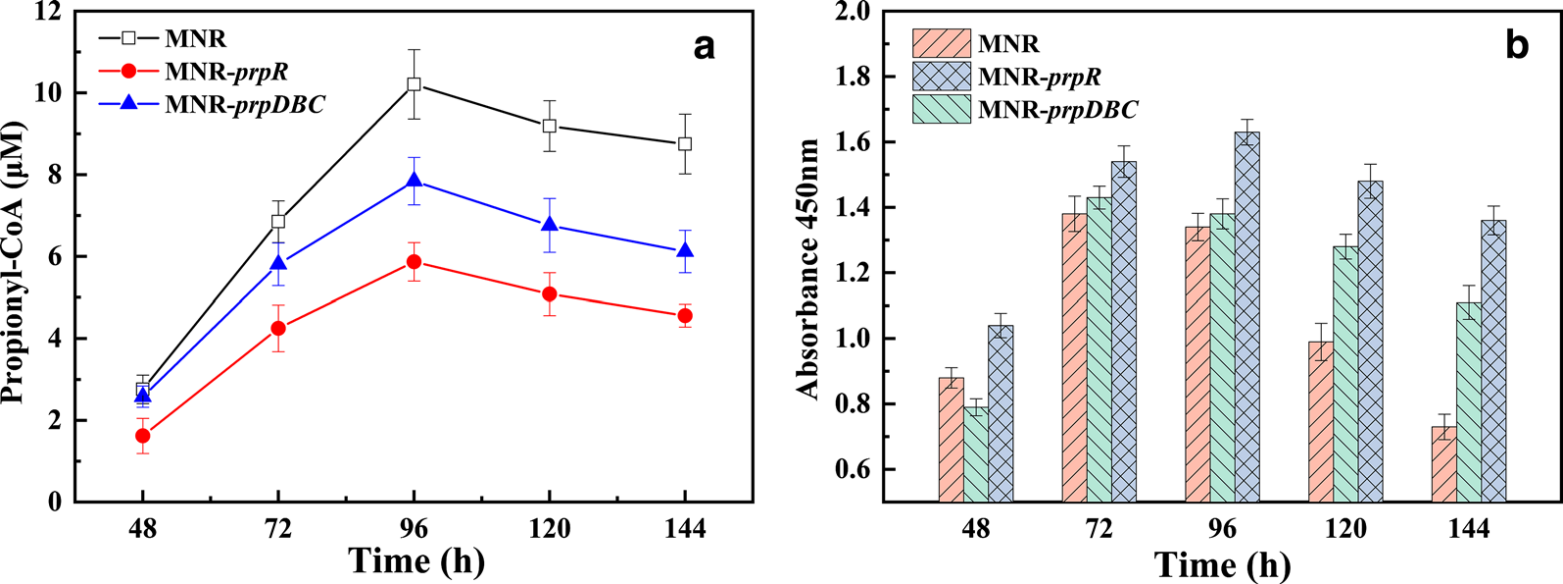
Intracellular propionyl-CoA level and cell viability of MNR, MNR-*prpR* and MNR-*prpDBC*. **a** Propionyl-CoA levels of strains in the biotransformation process. **b** Cell viability of strains in the biotransformation process. Error bars indicate the standard deviation from three independent experiments

The MNR, MNR-*prpR* and MNR-*prpDBC* intracellular propionyl-CoA level assays showed that the recombinant strains had similar trends to the parental strains. The overexpression of the *prpR* and *prpDBC* can effectively reduce the accumulation of propionyl-CoA in the middle and late stages of biotransformation (Fig. 3a), and the MNR-*prpR* has the lowest intracellular propionyl-CoA level. At 96 h, intracellular levels of propionyl-CoA in MNR-*prpR* and MNR-*prpDBC* (5.9 ± 0.5 and 7.8 ± 0.6 μM) were reduced by 43 ± 3% and 23 ± 4% compared to MNR (10.2 ± 0.9 μM). Moreover, the cell viability of recombinant strains was improved. The MNR-*prpR* always showed higher cell viability than MNR and MNR-*prpDBC* (Fig. 3b). The highest cell viability of MNR-*prpR* (1.6 ± 0.04) was 22 ± 1% higher than that of MNR (1.3 ± 0.04). Although the cell survival rates of the three strains decreased after 96 h, MNR-*prpR* and MNR-*prpDBC* showed a slower downward trend than MNR. At 144 h, the cell viability of MNR was only 53 ± 1% of the highest value, whereas the ratio of MNR-*prpR* was 83 ± 1%. In *M. tuberculosis*, MCC is essential for growth on propionate or cholesterol. Masiewicz et al. [26] confirmed that PrpR as a transcription factor is directly involved in the regulation of genes encoding the key enzymes of methylcitrate PrpD, PrpC and isocitrate lyase Icl1 cycles. The deletion of the prpR genes results in impaired growth in vitro on propionate or cholesterol as a sole carbon source. Similar results were obtained for non-pathogenic *M. smegmatis* [19].

The MCC plays an essential role in the detoxification of propionyl-CoA in mycobacteria. The overexpression of the *prp* operon can enhance the MCC, reduce the intracellular accumulation of propionyl-CoA and maintain cell viability. The reduction of propionyl-CoA also has a promoting effect on the side chain degradation of phytosterols, effectively improving the AD production capacity of MNR.

### The function of *glnR* in MNR

#### *glnR* expression repressed the transcription of *prpDBC*

**Fig. 4:**
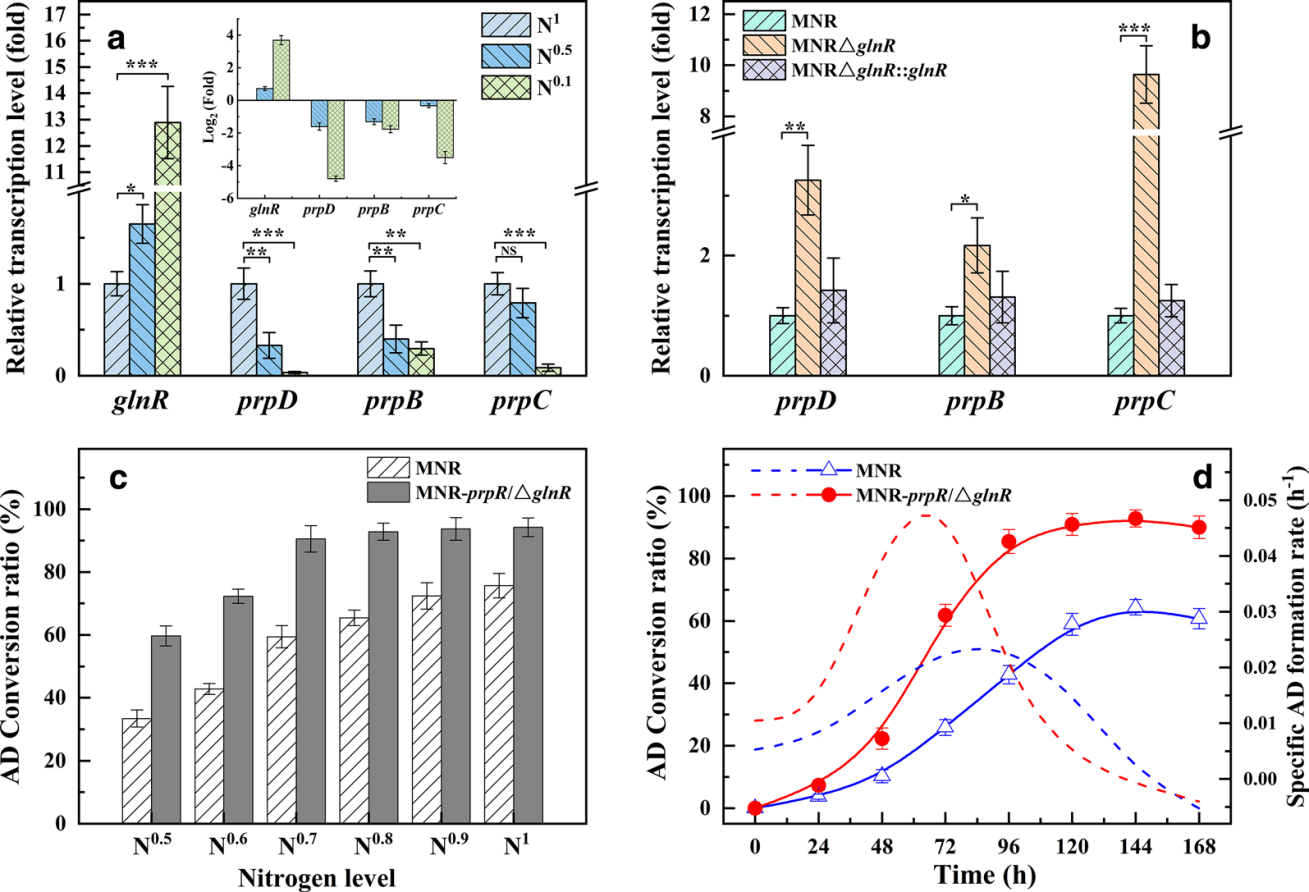
The function of *glnR* in MNR. **a** The transcription of *prpDBC* is responsive to nitrogen availability. **b** GlnR represses the transcription of *prpDBC* in MNR. **c** Optimization of the addition of nitrogen levels for AD production. **d** Time course of AD production by MNR-*prpR*/Δ*glnR* in low nitrogen levels. Error bars indicate the standard deviation from three independent experiments and the levels of statistical significance are indicated as follows: *p < 0.05; **p  < 0.01; ***p < 0.001

In the study of the effects of different nitrogen sources on the conversion of phytosterols by MNR, diammonium phosphate was found to be an excellent inorganic nitrogen source to replace yeast extracts. The optimum amount of (NH_4_)_2_HPO_4_ for use was 3.5 g/L, and the yield of AD decreased significantly with the decrease of (NH_4_)_2_HPO_4_ usage (data not shown). During the growth of *M. smegmatis*, nitrogen restriction increases the level of *glnR* transcription and inhibits the transcription of *prpDBC* involved in the MCC [36]. Inspired by this, the transcriptional levels of *glnR* and *prpDBC* in MNR were studied at different nitrogen source levels. As shown in Fig. 4a, the *glnR* transcription level increased 12.9 ± 1.4-fold at the nitrogen-limited level (N^0.1^), but a 96 ± 1% decrease for prpD, a 71 ± 2% decrease for prpB, and a 91 ± 1% decrease for prpC. At a low nitrogen source level (N^0.5^), the same trend was observed for *prp* operon. A *prpR* knockout (MNR*ΔglnR*) and a replenishing strain (MNR*ΔglnR*::glnR) were constructed and used in further confirming the effect of PrpR on the transcription of *prpDBC*. The transcription levels of *prpDBC* genes were studied under N^0.1^ conditions (Fig. 4b). Compared with MNR, the deletion of *glnR* resulted in increased *prpD*, *prpB* and *prpC* transcripts by 3.3 ± 0.6, 2.2 ± 0.5 and 9.6 ± 1.1 times, respectively.

The transcription levels of *prpDBC* in MNR*ΔglnR*::glnR were equivalent to that in MNR. These results further demonstrated that GlnR inhibited the transcription of *prpDBC* involved in the MCC in response to stress from low nitrogen sources. These findings were consistent with previous reports, the deletion of *glnR* alleviates the GlnR-mediated repression of *prpDBC* and increases the activity of the MCC (assimilation of propanoate and propionyl-CoA) [36].

#### Effects of deleting *glnR* on cell growth and biotransformation

The existing data indicate *glnR* is a global transcriptional regulator. In *Saccharopolyspora erythraea*, *glnR* can not only regulate nitrogen metabolism, but also regulate carbon metabolism [42] and homeostasis of intracellular phosphorus [43], and affects the synthesis of actinomycete antibiotics in *Streptomyces* [44]. Recent studies showed that GlnR inhibits the transcription of the *prp*DBC involved in the MCC at low nitrogen levels. The findings revealed a unique link between nitrogen metabolism and propionyl-CoA assimilation involved in fatty acid or cholesterol utilization [36]. The GlnR responds to low nitrogen source levels and inhibits *prpDBC*. PrpD, PrpB and PrpC are the core enzymes of MCC and play an important role in the metabolism of propionyl-CoA. The overexpression of *prp* operon in MNR*ΔglnR* affects the AD production of MNR. The *prpR* and *prpDBC* overexpression strains of MNR*ΔglnR* (MNR-*prpR*/Δ*glnR* and MNR-*prpDBC*/Δ*glnR*) were constructed, and the cell growth and biotransformation of different strains were studied at different levels of nitrogen sources. As shown in Table 2, all strains had low biomass and biotransformation rates under N^0.1^ conditions. With the increase nitrogen source level, the two indices of each strain increased significantly. At the same nitrogen source level, *glnR* deletion was beneficial to the transformation of phytosterols, and the overexpression of *prp* operon gene cluster can further enhance the transformation ability of MNR. Consistent with the above results, the overexpression of *prpR* in *glnR*-deleted strains was more effective than *prpDBC*. Under the conditions of N^0.1^, N^0.5^ and N^1^, the biotransformation rates of MNR-*prpR*/Δ*glnR* were 21.6 ± 2.4%, 57.7 ± 2.8% and 94.3 ± 3.4%, which were 1.6, 1.4 and 1.2 times higher than MNR (13.8 ± 2.8%, 42.4 ± 2.7 and 75.7 ± 3.5), respectively. These results indicated that the nitrogen source level is an important factor affecting the cell growth and AD production of MNR. At low nitrogen level, the deletion of *glnR* relieved the inhibition of GlnR on *prpDBC* and increased the metabolism of propionyl-CoA by MCC.

**Table 2:** Recombinant strain growth status and AD conversion ratio in different levels of nitrogen sources

Strains	N^0.1^ (0.35 g/L NH_42_PO_4_)		N^0.5^ (1.75 g/L NH_42_PO_4_)		N^1^ (3.5 g/L NH_42_PO_4_)	
	Final OD_600_	Max AD converted (%)	Final OD_600_	Max AD converted (%)	Final OD_600_	Max AD converted (%)
MNR	6.5 ± 0.2	13.8 ± 2.8	10.3 ± 0.3	42.4 ± 2.7	11.5 ± 0.6	75.7 ± 3.5
MNRΔglnR	7.0 ± 0.3	16.7 ± 2.3	10.8 ± 0.4	49.3 ± 2.3	10.8 ± 0.5	86.3 ± 3.7
MNR-prpDBC/ΔglnR	6.8 ± 0.2	17.3 ± 2.5	10.3 ± 0.3	52.3 ± 2.8	10.2 ± 0.5	91.7 ± 3.6
MNR-prpR/ΔglnR	6.7 ± 0.2	21.6 ± 2.4	10.8 ± 0.4	57.7 ± 2.8	11.2 ± 0.6	94.3 ± 3.4

#### Effect of different nitrogen sources levels on the AD production of MNR-*prpR*/Δ*glnR*

The impact of varying nitrogen source levels on the AD production of MNR and MNR-*prpR*/Δ*glnR* were investigated in a shake flask. The result was used in studying the feasibility of reducing the cost of nitrogen sources. The biotransformation rates of MNR and MNR-*prpR*/Δ*glnR* increased with nitrogen source level from N^0.5^ to N^1^ (Fig. 4c). The AD biotransformation rates of MNR-*prpR*/Δ*glnR* (90.6 ± 4.2%) under N^0.7^ condition was 1.2 times than that of MNR under N^1^ condition (75.7 ± 3.9%). The AD biotransformation rates of MNR-*prpR*/Δ*glnR* increased slightly with nitrogen content. The AD conversion rates of MNR-*prpR*/Δ*glnR* were 92.8 ± 2.7%, 93.7 ± 3.6% and 94.2 ± 3.0% under N^0.8^, N^0.9^ and N^1^ conditions, respectively. Therefore, in subsequent experiments, N^0.7^ conditions were selected for the investigation of the phytosterol biotransformation of MNR-*prpR*/Δ*glnR*. As shown in Fig. 4d, compared with MNR, MNR-*prpR*/Δ*glnR* had higher phytosterol biotransformation ability during fermentation. At 144 h, the highest biotransformation rate of MNR-*prpR*/Δ*glnR* was 92.8 ± 2.7%, which was 28.4 ± 1.0% higher than that of MNR (64.4 ± 2.5%). A comparison of the AD formation rate (*q*_*p*_) of MNR and MNRΔglnR*-prpR* showed that the highest *q*_*p*_ (0.045) of MNRΔglnR*-prpR* at 72 h was 2 times higher than the highest *q*_*p*_ (0.023) of MNR at 96 h. Moreover, the production time for the highest *q*_*p*_ for MNRΔglnR*-prpR* was shorter by 24 h. Therefore, the AD production performance of MNRΔglnR*-prpR* is better than that of MNR at the low nitrogen source level.

### A novel strategy for improving phytosterol biotransformation in low nitrogen levels by the transcriptional regulators PrpR and GlnR

**Fig. 5:**
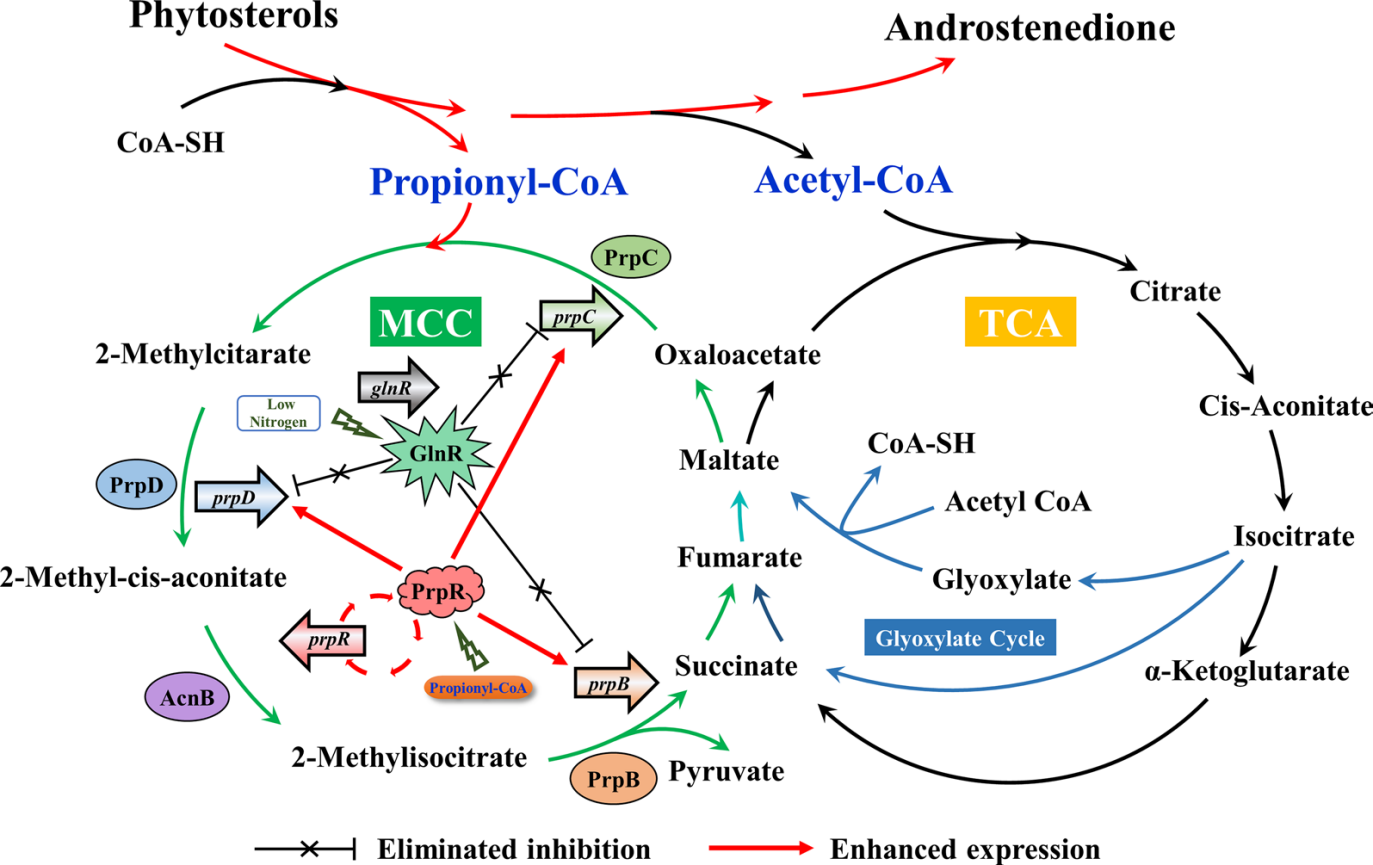
The regulatory mechanism of GlnR and PrpR on *prpDBC* involved in the methylcitrate cycle pathway of MNR

Our previous studies showed that the enhancement of the propionyl-CoA carboxylase gene in the MMC of propionyl-CoA metabolism could improve the ability of MNR to transform phytosterols. In the present study, based on the identification of *prp* operon and *glnR* in MNR, a novel strategy of improving phytosterol biotransformation in a low nitrogen level was established (Fig. 5). In MNR, the overexpression of the *prp* operon enhanced MCC to reduce the accumulation of propionyl-CoA and its endotoxic, thus, enabling the strains to maintain high activity to achieve efficient production of AD. The combination strategy of knocking out *glnR* and overexpressing *prpR* can eliminate the inhibitory effect of *glnR* on *prpDBC*, increase the low nitrogen adaptation ability of MNR and enable the MNR-*prpR*/Δ*glnR* to produce AD efficiently under low nitrogen sources.

## Conclusions

In this study, we identified the *prp* operon and the *glnR* in MNR and studied the regulatory effects of PrpR and GlnR on *prpD*, *prpB* and *prpC*. The enhancement of *prpR* and *prpDBC* can effectively reduce the accumulation of propionyl-CoA and enhance the phytosterol biotransformation ability of MNR. Besides, *glnR*-deficient strains can better adapt to a low nitrogen source environment and reduce nitrogen source consumption in AD production. These findings provide new insights into the enhancement of the production capacity of steroid precursors of mycobacteria and the reduction of the cost of using nitrogen sources.

## Data Availability

All data generated or analyzed in this study are included in the published article.

## Abbreviations

AD: androstenedione
9α-OH-AD: 9α-hydroxyandrostenedione
MNR: *Mycobacterium neoaurum*
MFT: *Mycobacterium fortuitum*
*prpC*: methylcitrate synthase gene
*prpD*: methylcitrate dehydratase/hydratase gene
*prpB*: methylisocitrate lyase gene
MMC: methyl citrate cycle
*prpR*: propionate regulator PrpR gene
*glnR*: nitrogen transcription regulator GlnR gene
CoA: coenzyme A
DCW: dry cell weight
ROS: reactive oxygen species
MCC: 2-methylcitrate cycle pathway
LB: Luria–Bertani
Kan^R^: kanamycin resistance
qRT-PCR: quantitative Reverse Transcription-PCR
mRNA: messenger RNA
PBS: phosphate-buffered saline
HPLC: high-performance liquid
AcnD: methylcitrate dehydratase
PrpF: methylaconitate cis–trans isomerase
AcnB: aconitase
